# Effectiveness of brief motivational interviewing for alcohol misuse: a systematic review of randomized controlled trials

**DOI:** 10.1016/j.abrep.2026.100727

**Published:** 2026-07-10

**Authors:** Mei-Hsin Ho, Jui-Hsiu Tsai, Chizimuzo T C Okoli, Rei-Mei Hong

**Affiliations:** a Department of Medical Research, Dalin Tzu Chi Hospital, Buddhist Tzu Chi, Medical Foundation, Chiayi, Taiwan; b Department of Psychiatry, Dalin Tzu Chi Hospital, Buddhist Tzu Chi Medical Foundation, Chiayi, Taiwan; c School of Medicine, Tzu Chi University, Hualien, Taiwan; d College of Nursing, University of Kentucky, Lexington, Kentucky, USA; e Department of Nursing, Chang Gung University of Science and Technology, Chiayi Campus, Chiayi, Taiwan

**Keywords:** Brief motivational interviewing, Motivational interviewing, Alcohol use disorder, Brief intervention, Behavioral intervention

## Abstract

**Background:**

Alcohol misuse remains a major global public health concern. Brief motivational interviewing (BMI) is a time-efficient, patient-centered intervention for reducing hazardous and harmful alcohol use.

**Methods:**

This systematic review synthesized evidence from 32 randomized controlled trials published between 2015 and 2025 evaluating BMI interventions for alcohol misuse in adults. Studies involving university students, emergency department patients, individuals with psychiatric or medical comorbidities, and socially vulnerable populations were included.

**Results:**

BMI was generally associated with reductions in alcohol consumption, binge drinking frequency, heavy drinking days, alcohol-related consequences, and Alcohol Use Disorders Identification Test (AUDIT) scores. Effects were more consistently observed among university students and emergency department populations, whereas findings were more variable among individuals with psychiatric comorbidity or social vulnerability. Technology-assisted and hybrid BMI approaches demonstrated short-term effectiveness comparable to traditional face-to-face delivery, although intervention effects frequently attenuated beyond 6–12 months.

**Conclusions:**

BMI appears to be a flexible and clinically meaningful intervention for reducing alcohol-related harm across diverse adult populations. However, long-term benefits were less consistently maintained, suggesting that ongoing support strategies, such as booster contacts, digital monitoring, or continued clinician involvement, may be important for sustaining behavioral change over time.

## Highlights

• Brief motivational interviewing reduced alcohol use and related harms.
• Evidence from 32 randomized controlled trials supported short-term benefits.
• Binge drinking and alcohol-related consequences consistently decreased.
• Effects were strongest in university and emergency department populations.
• Booster contacts may help sustain intervention effects over time.

## 1 Introduction

Harmful alcohol use, defined as alcohol consumption leading to psychological or physical harm (Reid et al., 1999), remains a major global public health concern, contributing substantially to morbidity, mortality, and broader social harm worldwide. According to the World Health Organization, alcohol consumption accounts for approximately 2.6 million deaths annually and represents about 4.7% of all deaths globally (World Health Organization, 2024). Alcohol-related problems range from hazardous and harmful drinking patterns to alcohol use disorders (AUDs), which collectively impose a substantial burden on healthcare systems and society through high healthcare utilization, psychiatric comorbidity, injury risk, social dysfunction, and reduced quality of life (Astrup & Estruch, 2019; Rehm and Shield, 2019a, Rehm and Shield, 2019b; GBD 2016 Alcohol Collaborators, 2018).

Despite the availability of evidence-based treatments, many individuals with hazardous or harmful drinking patterns do not seek specialized treatment, highlighting the importance of brief and accessible intervention strategies. Barriers to treatment engagement may include stigma, limited access to care, financial constraints, and low readiness to change (Kaner et al., 2017; Saitz, 2014). Furthermore, intensive long-term interventions are often difficult to implement in routine healthcare settings because of limited time and resource constraints (D'Onofrio & Degutis, 2010). Consequently, brief interventions have become a key strategy for addressing risky alcohol use during opportunistic clinical encounters (Kaner et al., 2018).

Motivational Interviewing (MI), originally introduced by Miller (1983) and subsequently refined by Miller and Rollnick, 1991, Miller and Rollnick, 2023, is a collaborative, person-centered counseling approach designed to strengthen an individual's intrinsic motivation and commitment to change by exploring and resolving ambivalence. MI has demonstrated effectiveness across a range of health behaviors, including smoking cessation, weight management, medication adherence, and alcohol reduction (Ekong & Kavookjian, 2016; Lundahl et al., 2013). Brief Motivational Interviewing (BMI) represents a time-limited adaptation of MI that retains its core principles while enhancing feasibility in routine clinical practice. Brief forms of MI were developed to increase the practicality of motivational approaches in healthcare settings, particularly primary care and emergency departments, where many individuals with hazardous or harmful drinking patterns present but do not seek specialized treatment (Kaner et al., 2009; D'Onofrio & Degutis, 2010). BMI is typically delivered through one or more brief sessions and incorporates personalized feedback, reflective listening, elicitation of change talk, and collaborative goal setting. The theoretical basis of BMI is broadly consistent with self-determination theory and other motivational models of behavior change, which posit that individuals are more likely to modify health behaviors when intrinsic motivation, self-efficacy, and autonomy are strengthened. Through reflective listening and the strategic elicitation of change talk, BMI aims to enhance readiness for change while reducing resistance to behavior modification (Magill et al., 2014; Miller & Rollnick, 2023).

Previous systematic reviews and meta-analyses have demonstrated that BMI may produce small to moderate reductions in alcohol consumption, binge drinking episodes, heavy drinking days, and alcohol-related consequences (Kaner et al., 2017, Kaner et al., 2018; Mun et al., 2023; Schwenker et al., 2023; Tanner-Smith & Lipsey, 2015). Effects appear particularly promising when delivered during “teachable moments,” such as after alcohol-related injuries or acute health events, when heightened risk awareness and perceived vulnerability may increase readiness for behavior change (D'Onofrio & Degutis, 2010). In recent years, technology-assisted BMI formats—including web-based programs, smartphone applications, text messaging, and hybrid models—have shown modest but meaningful reductions in alcohol consumption while substantially improving accessibility for younger adults and underserved populations (Bertholet et al., 2023; Muench et al., 2024; Riper et al., 2018). Many earlier reviews focused on specific subpopulations, particularly college students and emergency department patients (Carey et al., 2007; Tanner-Smith & Lipsey, 2015). Broader meta-analyses have demonstrated small-to-moderate reductions in alcohol consumption and alcohol-related consequences, with stronger effects observed during short-term follow-up and among individuals experiencing alcohol-related health events (Kaner et al., 2017, Kaner et al., 2018; Mun et al., 2023).

Nevertheless, important gaps remain in the literature. Few contemporary syntheses have comprehensively examined how BMI effectiveness varies according to intervention intensity, delivery modality, psychiatric comorbidity, social vulnerability, and technological adaptation within a single framework. In particular, the potential value of repeated contact—including multi-session delivery and booster follow-up contacts—for maintaining alcohol-related improvements over time remains insufficiently understood. Repeated contact may reinforce motivation, strengthen self-efficacy, provide opportunities to revisit goals, and help address relapse risk after the initial intervention period. Prior research has suggested that interventions incorporating repeated contact may contribute to more durable behavior change outcomes and improved maintenance of treatment gains (Hettema et al., 2005; Lundahl et al., 2010; Magill et al., 2019). Additionally, intervention responsiveness may vary according to population complexity. Individuals with substantial psychiatric comorbidity or social vulnerability may experience attenuated effects because greater clinical and psychosocial complexity can reduce engagement in behavior change and increase barriers to sustained alcohol reduction.

Therefore, this systematic review synthesized evidence from randomized controlled trials published between 2015 and 2025 evaluating BMI interventions for alcohol misuse in adults. The review aimed to assess effectiveness across heterogeneous populations, healthcare settings, and delivery modalities. We hypothesized that: (1) BMI would produce significant short-term reductions in alcohol consumption and alcohol-related harms across diverse adult populations; (2) BMI interventions incorporating repeated contact would demonstrate greater maintenance of alcohol-related improvements over time than interventions without reinforcement contacts; (3) intervention effects would be more modest among individuals with substantial psychiatric comorbidity or social vulnerability; and (4) technology-assisted and hybrid BMI formats would demonstrate short-term effectiveness broadly comparable to traditional face-to-face approaches, although long-term sustainability might be less consistent in minimally guided formats. By examining these hypotheses, this review aims to provide a contemporary synthesis of BMI effectiveness and identify factors associated with sustained intervention benefits across diverse adult populations.

## 2 Methods

### 2.1 Study design

This systematic review was conducted in accordance with the Preferred Reporting Items for Systematic Reviews and Meta-Analyses (PRISMA 2020) Statement guidelines (Page et al., 2021). The review protocol was prospectively registered in the International Prospective Register of Systematic Reviews (PROSPERO; Registration No. CRD420261353273).

The review question and eligibility criteria were structured according to the PICO framework (population, intervention, comparison, and outcomes). BMI was defined as a time-limited intervention based primarily on MI principles, including collaborative communication, exploration and resolution of ambivalence, enhancement of intrinsic motivation, and support of self-efficacy (Miller & Rollnick, 2023). Eligible interventions consisted of one to four sessions lasting approximately 15–60 min each. This threshold was selected to preserve the distinction between brief motivational interventions and more extended psychotherapeutic treatments. Interventions exceeding four sessions were excluded because they were considered extended psychotherapeutic approaches rather than brief interventions. Booster contacts were defined as additional reinforcement contacts delivered after the primary BMI session(s), including telephone calls, text-message reminders, smartphone prompts, telemonitoring, or brief reinforcement sessions intended to support maintenance of behavior change. Because many interventions combined multiple BMI sessions with booster contacts, both components were conceptualized as forms of repeated contact rather than being evaluated as independent intervention elements. Given the variability in intervention structures across studies, the review examined repeated-contact strategies, including multiple BMI sessions and booster contacts, as a secondary focus related to intervention sustainability.

### 2.2 Search strategy

A comprehensive literature search was conducted across four electronic databases: PubMed, CINAHL, PsycINFO, and Web of Science. Searches included studies published or electronically available between January 2015 and December 2025 to capture contemporary evidence and recent developments in telehealth and digitally assisted BMI interventions. The final database search was performed on January 31, 2026.

The review focused exclusively on adult populations (≥18 years). This restriction was applied to improve population comparability because alcohol interventions targeting adolescents are often prevention-oriented, whereas adult interventions more commonly target reduction of established hazardous or harmful drinking behaviors and alcohol-related consequences.

## Search terms

were developed using the PICO framework. The core search combined terms related to the intervention and condition as follows:

(motivational interviewing OR brief motivational interviewing OR brief intervention OR BMI) AND (alcohol OR drinking OR ethanol OR alcohol use disorder OR heavy drinking)

Full database-specific search strings, including MeSH terms and limits (English language; adults ≥18 years), are provided in Supplementary Material Table S1.

Title and abstract screening and full-text assessment were performed independently by two reviewers. Reference lists of included studies and relevant reviews were manually screened by the same two reviewers to identify additional eligible articles. Searches for ongoing or unpublished trials were conducted in ClinicalTrials.gov and the World Health Organization International Clinical Trials Registry Platform (ICTRP) to minimize publication bias.

### 2.3 Study selection

Studies were included if they met the following criteria:

1. Employed a randomized controlled trial design.
2. Included adults aged 18 years or older.
3. Included participants with hazardous alcohol use, harmful alcohol use, or alcohol use disorder (AUD). Hazardous alcohol use generally refers to drinking patterns associated with increased risk of future harm, whereas harmful alcohol use referred to alcohol-related harm already occurring. AUD referred to more severe patterns characterized by impaired control or diagnostic criteria consistent with DSM or ICD classifications.
4. Evaluated BMI interventions or brief MI–based brief interventions consisting of one to four sessions.
5. Used MI as the primary therapeutic component.
6. Included delivery formats such as individual counseling, group-based interventions, telephone counseling, telehealth delivery, digitally assisted approaches, or hybrid delivery formats combining clinician-delivered BMI with digital monitoring, messaging, or web-based support components.
7. Reported quantitative alcohol-related outcomes, including alcohol consumption, drinking frequency, heavy drinking days, binge drinking episodes, or Alcohol Use Disorders Identification Test (AUDIT) scores.

Studies were excluded if they met any of the following criteria:

1. Focused primarily on substances other than alcohol without separately reporting alcohol-specific outcomes.
2. Evaluated fully automated digital interventions lacking clinician-guided MI components, because such interventions may not adequately reflect core relational and interactive principles of MI.
3. Involved psychotherapeutic interventions (including MI) exceeding four sessions.
4. Used non-randomized designs, including observational studies, qualitative studies, case reports, protocols, conference abstracts, and review articles.
5. Involved participants younger than 18 years.

Discrepancies were resolved through discussion and, when necessary, consultation with a third reviewer.

### 2.4 Data extraction

Data extraction was conducted independently by two reviewers using a standardized extraction form developed for this review. Extracted study characteristics included author, publication year, country, clinical setting, sample size, and number of study arms.

Participant characteristics included mean age, sex distribution, alcohol use severity (hazardous use, harmful use, or alcohol use disorder), psychiatric or medical comorbidities, and indicators of social vulnerability. Social vulnerability indicators included homelessness, unstable housing, poverty, limited healthcare access, exposure to violence, or other forms of social marginalization reported in the included studies. Intervention characteristics included delivery modality (face-to-face, telephone, telehealth, digital, or hybrid), intervention format (individual or group), number and duration of sessions, use of repeated-contact strategies (including multiple BMI sessions and/or booster contacts), incorporation of technological components, type of interventionist, and assessment of MI fidelity. Comparator conditions were also extracted, including usual care, brief advice, assessment-only conditions, and educational materials.

Outcome variables included alcohol consumption measures (drinks per week, drinking frequency, heavy drinking days, and binge drinking episodes), AUDIT scores, alcohol-related consequences, and follow-up duration. Follow-up periods were categorized as short-term (≤3 months), medium-term (>3 to 6 months), and long-term (>6 months), with extended follow-up defined as ≥12 months. Follow-up duration was defined as the time elapsed since initiation of the BMI intervention, regardless of whether booster contacts or additional reinforcement sessions were delivered during the follow-up period. Discrepancies were resolved by consensus. Heavy drinking days generally referred to days exceeding standard daily drinking thresholds (commonly ≥4 drinks for women or ≥ 5 drinks for men), whereas binge drinking episodes referred to consumption reaching these thresholds within a brief time period (typically within approximately 2 h), according to study-specific definitions. Alcohol-related outcomes were primarily based on self-reported measures, although several studies also incorporated validated screening instruments such as the AUDIT.

### 2.5 Risk of Bias assessment

Methodological quality was assessed independently by two reviewers using the Cochrane Risk of Bias 2 (RoB 2) tool (Sterne et al., 2019). Five domains were evaluated: (1) bias arising from the randomization process; (2) bias due to deviations from intended interventions; (3) bias due to missing outcome data; (4) bias in outcome measurement; and (5) bias in selection of reported results. Each domain was rated as “low risk,” “some concerns,” or “high risk,” and an overall risk-of-bias judgment was assigned. Disagreements were resolved through discussion or consultation with a third reviewer. Results are presented in Table 1.

**Table 1:** Risk of Bias Assessment of Included Studies Using the Cochrane RoB 2 Tool.

Study	D1. Randomization Process	D2. Deviations from Intended Interventions	D3. Missing Outcome Data	D4. Measurement of the Outcome	D5. Selection of the Reported Result	Overall Risk of Bias	Key Concerns
Bogner et al., 2021	Low	Low	Some concerns	Low	Low	Some concerns	Moderate attrition at 12 months
Colby et al., 2018	Low	Low	Some concerns	Some concerns	Low	Some concerns	Self-report bias
Constant et al., 2021	Some concerns	Low	Some concerns	Some concerns	Low	Some concerns	Allocation concealment
Dunn et al., 2020	Low	Low	Low	Low	Low	Low	No major concerns identified
Fernandez et al., 2017	Low	Low	Low	Some concerns	Low	Some concerns	Reliance on self-reported drinking game frequency
Gaume et al., 2022	Low	Low	Low	Some concerns	Low	Some concerns	Self-reported outcomes
Gex et al., 2023	Some concerns	Some concerns	High	Some concerns	Low	High	High attrition (pilot)
Gupta et al., 2024	Some concerns	Low	Some concerns	Some concerns	Some concerns	Some concerns	Limited information on prespecified outcomes
Hammarberg et al., 2024	Low	Low	Some concerns	Some concerns	Low	Some concerns	Lack of blinding for self-reported behavioral outcomes
Harder et al., 2020	Low	Low	Some concerns	Some concerns	Low	Some concerns	Incomplete outcome reporting
Hasin et al., 2022	Low	Low	Low	Low	Low	Low	No major concerns identified
Hettema et al., 2018	Low	Some concerns	Some concerns	Some concerns	Low	Some concerns	Differentiation and fidelity across treatment doses
Hides et al., 2021	Some concerns	Low	Some concerns	Some concerns	Low	Some concerns	Potential measurement bias due to follow-up procedures
Jo et al., 2023	Some concerns	Some concerns	Low	Some concerns	Some concerns	Some concerns	Limited blinding information
Kahler et al., 2018	Low	Low	Some concerns	Low	Low	Some concerns	Minor long-term attrition
Kuwabara et al., 2022	Some concerns	Low	Some concerns	Some concerns	Low	Some concerns	Cluster RCT reporting issues
Martín-Pérez et al., 2019	Low	Some concerns	Some concerns	Some concerns	Low	Some concerns	Potential group contamination
Mcqueen et al., 2015	Some concerns	Low	Low	Some concerns	Low	Some concerns	Performance bias in general hospital setting
Meinzer et al., 2021	Low	Low	Some concerns	Some concerns	Low	Some concerns	Comorbidity (ADHD) influence on retention
Mello et al., 2016	Low	Low	Low	Some concerns	Low	Some concerns	Self-reported data
Monti et al., 2016	Low	Low	Some concerns	Low	Low	Some concerns	Minor differential attrition
Oddo et al., 2021	Low	Low	Some concerns	Low	Low	Some concerns	Missing outcome data concerns
Parry et al., 2023	Low	Low	Low	Some concerns	Low	Some concerns	Potential deviations from intended intervention
Pedrelli et al., 2020	Low	Some concerns	Some concerns	Some concerns	Low	Some concerns	Potential influence of depression on outcome measurement
Rhodes et al., 2015	Low	Low	High	Some concerns	Low	High	High attrition in vulnerable group
Satre et al., 2016	Low	Low	Some concerns	Low	Low	Some concerns	Missing outcome data at long-term follow-up
Sinha et al., 2022	Some concerns	Low	Some concerns	Some concerns	Some concerns	Some concerns	Fidelity reporting
Soares & Vargas, 2020	Some concerns	Some concerns	Some concerns	Some concerns	Some concerns	Some concerns	Limited information on randomization procedures
Staton et al., 2025	Low	Low	Some concerns	Some concerns	Low	Some concerns	Limited information on prespecified analysis plan
Stein et al., 2021	Low	Low	Low	Low	Low	Low	No major concerns identified
Thompson Jr et al., 2017	Some concerns	Some concerns	High	Some concerns	Low	High	High attrition
Tucker et al., 2024	Low	Low	Some concerns	Some concerns	Low	Some concerns	Potential deviations from intended intervention

Note: Risk of bias was assessed using the Cochrane Risk of Bias 2 (RoB 2) tool. Studies were evaluated across five domains: (D1) bias arising from the randomization process, (D2) bias due to deviations from intended interventions, (D3) bias due to missing outcome data, (D4) bias in measurement of the outcome, and (D5) bias in selection of the reported result. Overall risk of bias judgments followed the RoB 2 algorithm, whereby any domain rated as high risk resulted in overall high risk of bias, and any domain rated as some concerns (with no high risk) resulted in overall some concerns. Abbreviations: ADHD: Attention-Deficit/Hyperactivity Disorder; HCV: Hepatitis C Virus; HIV: Human Immunodeficiency Virus; IPV: Intimate Partner Violence; TBI: Traumatic Brain Injury; ED: Emergency Department.

### 2.6 Data synthesis

A meta-analysis was not conducted because of substantial clinical and methodological heterogeneity across studies, including differences in participant populations, intervention characteristics, delivery modalities, comparator conditions, outcome measures, and follow-up duration.

Findings were synthesized narratively using a structured approach organized according to pre-specified domains derived from the review objectives and anticipated sources of heterogeneity identified in previous BMI literature. These domains included:

(1) Population type (e.g., university students, emergency department patients, individuals with psychiatric comorbidity, individuals with chronic medical conditions including HIV-related populations, and socially vulnerable groups);
(2) Intervention modality and delivery format (face-to-face, telehealth, digital, and hybrid approaches); and
(3) Repeated-contact characteristics (single-contact interventions, single-contact interventions with booster support, and interventions incorporating repeated contact through multiple sessions, booster contacts, or both).

Because multiple-session interventions and booster contacts frequently co-occurred, their independent effects could not be reliably distinguished across studies. Accordingly, synthesis focused on the broader concept of repeated contact as a potential mechanism supporting maintenance of behavior change. Patterns of effectiveness were examined across short-term (≤3 months), medium-term (>3–6 months), and long-term (>6 months) follow-up periods. Any descriptive summaries presented in the narrative synthesis or supplementary tables were derived from qualitative extraction of reported study findings and are intended solely to illustrate overall patterns across studies. These summaries should not be interpreted as pooled effect estimates, meta-analytic findings, or formal quantitative measures of treatment effectiveness.

### 2.7 Certainty of evidence assessment

The overall certainty of evidence for the major comparison domains identified in the narrative synthesis was assessed using the Grading of Recommendations Assessment, Development and Evaluation (GRADE) framework (Guyatt, Oxman, Schünemann, Tugwell, & Knottnerus, 2011). Randomized controlled trials were initially rated as high-certainty evidence and were downgraded when concerns were identified regarding risk of bias, inconsistency, indirectness, imprecision, or publication bias. Because quantitative pooling was not performed, GRADE assessments were based on the overall consistency, direction, and methodological quality of findings across included studies rather than pooled effect estimates. Certainty ratings were categorized as high, moderate, low, or very low.

## 3 Results

**Fig. 1:**
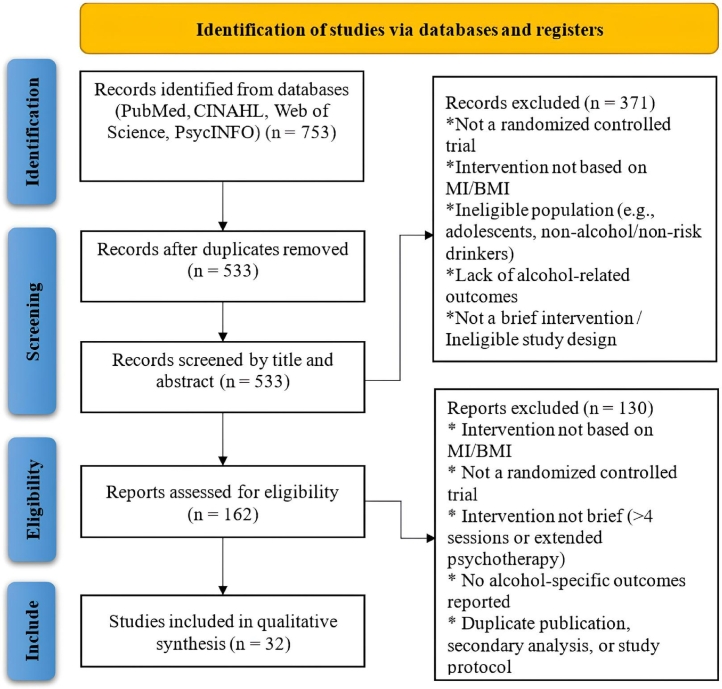
Flow diagram of study selection according to the PRISMA guidelines.

A total of 753 records were identified through database searches. After the removal of 220 duplicate records, 533 records underwent title and abstract screening, of which 371 were excluded. Full-text review was conducted for 162 articles, resulting in the exclusion of an additional 130 studies due to ineligible intervention type (*n* = 68), non-randomized design (*n* = 31), lack of alcohol-specific outcomes (*n* = 19), or inclusion of participants younger than 18 years (*n* = 12). Ultimately, 32 randomized controlled trials (RCTs) were included in the final synthesis. The study selection process is presented in Fig. 1.

### 3.1 Study and intervention characteristics

Across the 32 included RCTs, sample sizes ranged from 60 to 623 participants per study (median = 167; total participants = 6543). Most studies (*n* = 29) employed two-arm randomized designs (including one cluster-randomized trial), whereas three studies used multi-arm designs. Comparator conditions typically included usual care, brief advice, assessment-only conditions, or educational materials.

The included studies encompassed heterogeneous adult populations with hazardous alcohol use, harmful alcohol use, or alcohol use disorder, as defined in the eligibility criteria. Because several trials targeted intersectional populations meeting criteria for multiple subgroups, the cross-cutting analysis yielded a total of 37 population classifications across the 32 unique studies. These classifications primarily comprised university and emerging adult drinkers (*n* = 10), emergency department or hospital-based medical populations (*n* = 9), and individuals with psychiatric comorbidities or chronic medical conditions, including HIV infection (n = 10).

HIV and psychiatric populations were grouped for descriptive purposes because both frequently involve substantial medical and psychosocial complexity that may influence intervention responsiveness and follow-up adherence. Psychiatric comorbidities included depression, anxiety disorders, post-traumatic stress disorder, and severe mental illness across included studies. Additional populations included socially vulnerable groups (*n* = 3), and workplace, community, primary care, or general outpatient samples (*n* = 5). Socially vulnerable groups generally included individuals experiencing unstable housing, poverty, unemployment, or limited access to healthcare services. Detailed study characteristics are summarized in Table 2.

**Table 2:** Characteristics of Included Studies Evaluating Brief Motivational Interviewing (BMI) for Alcohol Use Reduction.

Study	Country	Population / Setting	Severity Category	Sample Size (Total / BMI)	Mean Age (years)	Study Design	Comparator Condition	Follow-up Time Points (months)	Primary Alcohol Outcomes	Attrition at Final Follow-up (%)
Bogner et al., 2021	USA	Adults with traumatic brain injury (TBI rehabilitation)	Hazardous /Risky Drinking	94 / 47	42.5	2-arm RCT	Educational intervention	3, 6, 12	Drinking frequency, binge drinking	14
Colby et al., 2018	USA	University students (underage)	Hazardous /Risky Drinking	172 / 86	20.1	2-arm RCT	Assessment-only	1, 3, 6	Drinks/week, binge episodes	18
Constant et al., 2021	Brazil	Outpatients (telehealth)	Harmful Drinking	114 / 57	39.4	2-arm RCT	Standard counseling	3, 6	Alcohol consumption	21
Dunn et al., 2020	USA	Mandated college students	Heavy Drinking	162 / 81	19.8	2-arm RCT	Alcohol education	1, 6	Heavy drinking days	9
Fernandez et al., 2017	USA	College students (drinking game participants)	Hazardous/Risky Drinking	134 / 68	19.7	2-arm RCT	Assessment-only	1, 3	Drinking game frequency, consumption	12
Gaume et al., 2022	Switzerland	Emergency department patients (alcohol intoxication)	Harmful Drinking	344 / 172	24.8	2-arm RCT	Standard care	3, 6, 12	Heavy drinking episodes	11
Gex et al., 2023	USA	Emerging adults (community)	Hazardous	60 / 30	22.3	Pilot 2-arm RCT	Assessment-only	1, 3	Drinks/week, consequences	38
Gupta et al., 2024	India	Adults with opioid use disorder (psychiatric comorbidity)	Alcohol Use Disorder (AUD)	120 / 60	35.7	2-arm RCT	Usual care	3, 6	AUDIT, drinking frequency	24
Hammarberg et al., 2024	Sweden	High-risk clinic outpatients	Heavy Drinking	210 / 105	44.2	2-arm RCT	Standard brief advice	6, 12	Weekly consumption, binge drinking frequency	15
Harder et al., 2020	Kenya	Community adults	Harmful Drinking	300 / 150	33.1	2-arm RCT	Usual care	3, 6	Alcohol consumption	19
Hasin et al., 2022	USA	People living with HIV (HIV primary care)	Alcohol Use Disorder (AUD)	139 / 46 †	47.3	3-arm RCT	Brief advice	6, 12	AUDIT score, heavy drinking days	10
Hettema et al., 2018	USA	Hospital outpatients	Hazardous /Risky Drinking	240 / 120	38.9	2-arm RCT	Treatment as usual	3, 6, 12	Heavy drinking days (HDD), AUDIT	17
Hides et al., 2021	Australia	Emergency department patients (alcohol-related injury)	Hazardous	331 / 166	21.7	2-arm RCT	Telephone support	1, 6, 12	Binge drinking frequency	23
Jo et al., 2023	South Korea	Internal medicine outpatients	Hazardous/Risky Drinking	154 / 77	48.2	2-arm RCT	Brief advice	3, 6	AUDIT scores	16
Kahler et al., 2018	USA	Men who have sex with men living with HIV	Heavy Drinking	180 / 90	41.6	2-arm RCT	Educational control	6, 12	Drinks/week, harms	15
Kuwabara et al., 2022	Japan	Workplace employees	Hazardous	277 / 138	43.5	Cluster RCT	Health education	3, 6	AUDIT scores	20
Martín-Pérez et al., 2019	Spain	Risky college drinkers	Hazardous	89 / 45	20.5	3-arm RCT	Group CBT	3, 6	Alcohol consumption	22
Mcqueen et al., 2015	UK	General hospital inpatients	Harmful Drinking	146 / 73	46.8	2-arm RCT	Information leaflet	6, 12	Alcohol intake (units/week)	19
Meinzer et al., 2021	USA	College students with ADHD	Hazardous	88 / 44	20.3	2-arm RCT	Alcohol education	3, 6	Binge drinking frequency, harms	14
Mello et al., 2016	USA	Injured emergency department patients	Harmful Drinking	225 / 113	31.4	2-arm RCT	Standard care	3, 12	Heavy drinking days	12
Monti et al., 2016	USA	Emergency department patients	Hazardous	184 / 92	23.9	2-arm RCT	Educational materials	3, 6, 12	Alcohol use, sexual risk	17
Oddo et al., 2021	USA	College students with ADHD	Hazardous	109 / 55	21.3	2-arm RCT	Standard BMI	3, 6	Binge drinking frequency	13
Parry et al., 2023	South Africa	People living with HIV on ART	Heavy Drinking	623 / 312	39.8	2-arm RCT	Standard HIV care	6, 12	Alcohol consumption	11
Pedrelli et al., 2020	USA	College students with depression	Heavy Drinking	94 / 47	20.8	2-arm RCT	CBT only	3, 6	Drinks/week, binge	26
Rhodes et al., 2015	USA	Emergency department patients (IPV + heavy drinking)	Harmful Drinking	600 / 300	31.7	2-arm RCT	Resource referral	3, 6, 12	Alcohol use	34
Satre et al., 2016	USA	Outpatients with depression	Hazardous	307 / 154	44.5	2-arm RCT	Usual care	3, 6, 12	Drinking frequency	16
Sinha et al., 2022	India	Outpatients with mood disorders	Hazardous	120 / 60	36.4	2-arm RCT	Standard psychiatric care	3, 6	AUDIT score, binge drinking frequency	25
Soares & Vargas, 2020	Brazil	Community adults	Hazardous/Risky Drinking	118 / 59	38.6	2-arm RCT	Educational group	3	Alcohol use and motivation to change	28
Staton et al., 2025	Tanzania	Emergency department patients	Harmful Drinking	484 / 161†	35.2	Multi-arm Adaptive RCT	Brief intervention only	6, 12	Alcohol consumption	18
Stein et al., 2021	USA	People with HIV-HCV co-infection	Heavy Drinking	114 / 57	51.1	2-arm RCT	Brief advice	6, 12	Drinks/week	9
Thompson Jr et al., 2017	USA	Homeless young adults	Hazardous	61 / 31	21.5	2-arm RCT	Assessment-only	1, 3	Alcohol consumption	36
Tucker et al., 2024	USA	Emerging adults experiencing homelessness	Hazardous	150 / 75	22.7	2-arm RCT	Usual services	6, 12, 24	Alcohol consumption	21

Note: Severity categories were assigned descriptively to facilitate comparison across heterogeneous study populations. Follow-up time points are reported in months after intervention initiation and do not necessarily represent the final intervention contact because some studies included repeated-contact strategies (e.g., multiple BMI sessions, booster contacts, or technology-assisted reinforcement).

† BMI group size was estimated because allocation across intervention arms was not explicitly reported. Abbreviations: ADHD = Attention-Deficit/Hyperactivity Disorder; ART = Antiretroviral Therapy; AUD = Alcohol Use Disorder; AUDIT = Alcohol Use Disorders Identification Test; BMI = Brief Motivational Interviewing; CBT = Cognitive Behavioral Therapy; ED = Emergency Department; HCV = Hepatitis C Virus; HIV = Human Immunodeficiency Virus; IPV = Intimate Partner Violence; RCT = Randomized Controlled Trial; TBI = Traumatic Brain Injury.

BMI interventions were generally brief, most commonly consisting of one to three sessions lasting 20–60 min. Nineteen studies incorporated more than one intervention contact, including multi-session interventions and single-session interventions supplemented by structured booster contacts. A booster contact was defined as any planned follow-up session or communication delivered after completion of the primary BMI session(s) to reinforce motivation, self-monitoring, or behavior change maintenance. Most studies (*n* = 30) primarily used individual delivery formats, whereas two incorporated group-based BMI approaches.

Regarding delivery modality, 25 studies primarily used face-to-face delivery, five incorporated hybrid or technology-assisted formats, and two primarily used telephone-based delivery. Among these, 17 studies included structured or planned booster contacts, including one study that provided optional booster follow-up, the number of booster contacts ranged from 1 to 12, with a median of 3, and most studies (*n* = 12) used 2–4 booster contacts. Boosters were most commonly delivered via telephone calls, text messaging/SMS, or brief smartphone prompts. Detailed intervention characteristics are presented in Table 3. Motivational interviewing fidelity assessment was reported in 22 of the 32 studies (68.8%), whereas 10 studies did not provide formal fidelity evaluation procedures.

**Table 3:** Characteristics of Brief Motivational Interviewing (BMI) Interventions Included in the Review.

Study	Delivery Mode	Format	Sessions (n)	Duration per Session	Booster Contact	Supplementary Support Component	Interventionist	Repeated-Contact Classification	MI Fidelity Assessment
Bogner et al., 2021	Face-to-face	Individual	1	45–60 min	No	None	Counselor	Single-contact	Yes
Colby et al., 2018	Face-to-face	Individual	1	45 min	No	Personalized feedback report	Psychologist	Single-contact	Yes
Constant et al., 2021	Telephone	Individual	2	30 min	Yes	Telehealth counseling	Counselor	Repeated-contact	No
Dunn et al., 2020	Face-to-face	Individual	1	60 min	No	None	Graduate clinician	Single-contact	Yes
Fernandez et al., 2017	Face-to-face	Individual	1	45–50 min	No	Personalized feedback graphics	Research assistant	Single-contact	Yes
Gaume et al., 2022	Face-to-face	Individual	1	20–30 min	Optional	None	ED clinician	Single-contact + Booster	Yes
Gex et al., 2023	Hybrid	Individual	3	20–30 min	Yes	Smartphone application + reminders	Therapist	Repeated-contact	Yes
Gupta et al., 2024	Face-to-face	Individual	4	45 min	Yes	None	Psychiatrist/Psychologist	Repeated-contact	No
Hammarberg et al., 2024	Face-to-face	Individual	1–2	30–45 min	Yes	None	Specialized nurse	Repeated-contact	Yes
Harder et al., 2020	Face-to-face	Individual	1	30 min	No	None	Trained lay counselor	Single-contact	Yes
Hasin et al., 2022	Face-to-face	Individual	2–4	30–45 min	Yes	Smartphone application (HealthCall)	Addiction counselor	Repeated-contact	Yes
Hettema et al., 2018	Hybrid	Individual	2	45 min	Yes	Telephone booster support	Trained clinician	Repeated-contact	Yes
Hides et al., 2021	Telephone	Individual	2	30 min	Yes	Telephone support	Counselor	Repeated-contact	No
Jo et al., 2023	Face-to-face	Individual	1	15–20 min	No	None	Physician	Single-contact	No
Kahler et al., 2018	Face-to-face	Individual	2	60 min	Yes	None	Clinical psychologist	Repeated-contact	Yes
Kuwabara et al., 2022	Face-to-face	Individual	1	20–30 min	No	Educational materials	Occupational health staff	Single-contact	No
Martín-Pérez et al., 2019	Face-to-face	Group	4	60–90 min	No	None	Psychologist	Repeated-contact	Yes
Mcqueen et al., 2015	Face-to-face	Individual	1	20–30 min	No	None	Project practitioner	Single-contact	Yes
Meinzer et al., 2021	Face-to-face	Individual	1	50–60 min	No	None	Clinical psychology student	Single-contact	Yes
Mello et al., 2016	Face-to-face	Individual	1	30 min	No	None	ED interventionist	Single-contact	Yes
Monti et al., 2016	Face-to-face	Individual	1	45 min	Yes	Telephone booster	Counselor	Single-contact + Booster	Yes
Oddo et al., 2021	Face-to-face	Individual	2	45 min	No	None	Therapist	Repeated-contact	Yes
Parry et al., 2023	Face-to-face	Individual	2	30 min	Yes	SMS reminders	Counselor	Repeated-contact	No
Pedrelli et al., 2020	Face-to-face	Individual	2	45–60 min	Yes	None	Psychologist	Repeated-contact	Yes
Rhodes et al., 2015	Face-to-face	Individual	2	30–45 min	Yes	Referral support tools	Social worker	Repeated-contact	No
Satre et al., 2016	Face-to-face	Individual	1	45 min	No	None	Behavioral health clinician	Single-contact	Yes
Sinha et al., 2022	Face-to-face	Individual	3	30–45 min	Yes	None	Psychiatrist	Repeated-contact	No
Soares & Vargas, 2020	Face-to-face	Group	4	60 min	No	None	Community facilitator	Repeated-contact	No
Staton et al., 2025	Hybrid	Individual	1–4	20–40 min	Yes	Mobile follow-up support	Counselor	Repeated-contact	Yes
Stein et al., 2021	Hybrid	Individual	2	30 min	Yes	Telephone monitoring	Addiction specialist	Repeated-contact	Yes
Thompson Jr et al., 2017	Face-to-face	Individual	1	30 min	No	None	Outreach counselor	Single-contact	No

Note: Repeated-contact classifications were assigned descriptively based on the intervention structure reported in each study. Supplementary support components are presented for descriptive purposes only and were not analyzed as separate intervention categories.

Abbreviations: BMI = Brief Motivational Interviewing; ED = Emergency Department; MI = Motivational Interviewing; SMS = Short Message Service.

### 3.2 Risk of Bias assessment

The risk of bias assessment is summarized in Table 1. Overall, 3 studies were rated as low risk of bias, 26 were judged to have some concerns, and 3 were rated as high risk of bias. High-risk ratings were primarily attributable to substantial missing outcome data and attrition in socially vulnerable or pilot-study populations. The most common concerns involved missing outcome data and outcome measurement, particularly because most studies relied on self-reported alcohol consumption measures and were unable to blind participants to behavioral intervention allocation.

Across studies, attrition rates ranged from 9% to 38% (median = 17.5%). Concerns related to deviations from intended interventions were generally limited and primarily reflected insufficient reporting of intervention fidelity or treatment differentiation procedures. No study was judged to be at high risk of bias in the domains of randomization or selection of the reported result.

### 3.3 Findings related to hypothesis 1: Overall effectiveness of BMI interventions

Overall, 24 of the 32 (75%) included studies reported statistically significant short-term improvements in at least one alcohol-related outcome favoring BMI. Positive effects were most commonly observed in measures of binge drinking frequency, heavy drinking days, alcohol-related consequences, and hazardous drinking severity as assessed by instruments such as the AUDIT.

Across studies demonstrating significant intervention effects, BMI was generally associated with reductions in risky drinking behaviors and alcohol-related harms during short-term follow-up periods. Improvements were most consistently observed within the first one to six months after intervention delivery. Several studies also reported reductions in overall alcohol consumption; however, findings for total alcohol volume were less consistent than those observed for binge drinking frequency and alcohol-related consequences.

Most primary alcohol-related outcomes were assessed during short-term (≤3 months) or medium-term (>3–6 months) follow-up periods, whereas relatively few studies included assessments extending beyond 12 months. Where effect sizes were reported, intervention effects were generally small to moderate in magnitude. Across the evidence base, beneficial effects were typically strongest during short-term follow-up and tended to attenuate over longer observation periods. Taken together, the available evidence suggests that BMI may contribute to short-term reductions in hazardous alcohol use and alcohol-related harms among adults.

### 3.4 Findings related to hypothesis 2: Repeated contact and maintenance of intervention effects

Follow-up duration was defined according to the time elapsed since the initial BMI session, regardless of whether booster contacts were subsequently delivered. Consequently, six-month follow-up outcomes reflect assessments conducted approximately six months after intervention initiation rather than six months after the final booster contact. Among the included studies, 27 reported short-term follow-up outcomes (≤3 months), 21 reported medium-term outcomes (>3 to 6 months), and 13 included long-term follow-up assessments (>6 months). Initial reductions in alcohol consumption frequently weakened after 6–12 months, particularly for total weekly alcohol consumption outcomes. Among alcohol-related outcomes, sustained reductions were more commonly observed for binge drinking frequency and alcohol-related consequences than for overall alcohol consumption volume. In contrast, studies without booster contacts more frequently demonstrated attenuation or non-significant effects during extended follow-up periods. Technology-assisted interventions that incorporated repeated contacts, text-message boosters, smartphone monitoring, or web-based self-monitoring generally appeared to demonstrate more sustained effects than minimally guided single-contact approaches.

Studies incorporating repeated contact, whether through multiple BMI sessions, booster follow-up contacts, or a combination of both, more frequently reported maintenance of alcohol-related improvements beyond 6 months compared with studies that did not include reinforcement contacts (Gupta et al., 2024; Hasin et al., 2022; Hettema et al., 2018; Staton et al., 2025; Tucker et al., 2024). Therefore, findings should be interpreted as reflecting the potential value of repeated contact rather than the superiority of any specific intervention component. The overall findings support Hypothesis 2 by suggesting that repeated contact may contribute to the maintenance of alcohol-related improvements over time. Although repeated-contact interventions generally demonstrated better maintenance of outcomes, long-term effectiveness remained heterogeneous across populations and intervention formats. Detailed outcome trajectories across follow-up periods are summarized in Supplementary Material Table S2.

### 3.5 Findings related to hypothesis 3: Population complexity and intervention responsiveness

Intervention effectiveness varied substantially according to participant characteristics and population complexity, as summarized in Table 4. Because several studies targeted intersectional populations meeting criteria for multiple subgroups, participant cohorts were cross-classified across multiple categories, resulting in 37 population classifications derived from the 32 unique randomized controlled trials.

**Table 4:** Summary of BMI Effectiveness by Population Type.

Population Type	No. of Studies	Short-term Effectiveness	Main Outcomes	Typical Pattern of Improvement	Long-term Maintenance (>6–12 months)	Key Findings
University / Emerging adult students	10	Most studies (9/10)	Binge drinking frequency, heavy drinking days, alcohol-related consequences	Consistent reductions in binge drinking frequency and alcohol-related consequences	Moderate; attenuation observed in some studies beyond 12 months	This population demonstrated the most consistent improvements across studies. One study involving participants with ADHD reported attenuated effects, suggesting that co-occurring clinical conditions may influence intervention responsiveness
Emergency Department / Hospital-based medical populations	9	Most studies (8/9)	Heavy drinking days, binge drinking frequency, alcohol-related harms	Reductions in heavy drinking days and alcohol-related harms	Moderate; maintenance was more frequently reported in studies incorporating repeated contact	Consistent short-term improvements were observed across most studies, although interpretation of long-term outcomes was limited in some studies because of attrition
Psychiatric / Medical comorbidities (including HIV)	10	Most studies (8/10)	AUDIT scores, heavy drinking frequency, alcohol consumption	Modest improvements in AUDIT scores and drinking outcomes	Variable	Findings were heterogeneous, and intervention effects were generally less consistent than those observed among university students or emergency department populations
Socially vulnerable populations	3	Mixed findings (2/3)	Alcohol use, alcohol-related consequences	Small and inconsistent improvements	Limited maintenance	High attrition, unstable living conditions, and competing psychosocial demands frequently reduced intervention engagement and follow-up participation
Workplace / Community / General outpatient populations	5	Mixed findings (3/5)	AUDIT scores, alcohol consumption quantity	Modest reductions in alcohol consumption and AUDIT scores	Variable	Modest improvements were observed, although findings were generally less consistent than those reported in university or emergency department populations

Note: Some studies were classified into more than one population category; therefore, the total number of population classifications (37) exceeds the number of included studies (32 randomized controlled trials). *Short-term effectiveness* refers to statistically significant improvements favoring BMI in at least one primary alcohol-related outcome within 6 months of follow-up. Findings represent a descriptive narrative synthesis and should not be interpreted as pooled effect estimates or formal comparative analyses. Abbreviations: ADHD = Attention-Deficit/Hyperactivity Disorder; AUDIT = Alcohol Use Disorders Identification Test; BMI = Brief Motivational Interviewing; HIV = Human Immunodeficiency Virus.

Among university students and emerging adult drinkers (*n* = 10 population classifications), BMI demonstrated the most consistent and favorable outcomes. Most studies in this subgroup reported significant short-term reductions in binge drinking frequency, heavy drinking days, and alcohol-related consequences (Colby et al., 2018; Dunn et al., 2020; Fernandez et al., 2017; Meinzer et al., 2021; Oddo et al., 2021; Pedrelli et al., 2020). These findings suggest that BMI may be particularly effective among younger adults who engage in hazardous or episodic binge drinking and who may be more receptive to behavior-change interventions during transitional developmental stages. Although attenuated effects were observed in some clinically elevated subgroups, including one study involving participants with attention-deficit/hyperactivity disorder (ADHD), overall intervention responsiveness remained favorable within this population. A somewhat more heterogeneous pattern was observed among emergency department and hospital-based medical populations (*n* = 9 classifications). Studies conducted in acute care settings frequently reported short-term reductions in heavy drinking days and alcohol-related harms following BMI delivery (Gaume et al., 2022; Hides et al., 2021; McQueen et al., 2015; Mello et al., 2016; Monti et al., 2016; Rhodes et al., 2015; Staton et al., 2025). Interventions were commonly delivered shortly after alcohol-related injury, crisis presentation, or medical contact, circumstances that may increase receptivity to behavioral intervention. However, maintenance of treatment effects was less consistent over longer follow-up periods, and several studies reported substantial attrition, particularly among socially or medically vulnerable participants.

Findings among individuals with psychiatric comorbidities or chronic medical conditions, including HIV infection and other complex health conditions (*n* = 10 classifications), were considerably more variable. Several studies demonstrated significant short-term improvements, primarily reflected by modest reductions in AUDIT scores (typically 2–5 points) and decreases in heavy drinking frequency rather than large reductions in overall alcohol consumption volume (Gex et al., 2023; Gupta et al., 2024; Hammarberg et al., 2024; Hasin et al., 2022; Hettema et al., 2018; Kahler et al., 2018; Parry et al., 2023; Satre et al., 2016; Sinha et al., 2022; Stein et al., 2021). Nevertheless, intervention effects within this subgroup were generally less consistent than those observed among university-based populations. Sustained reductions in alcohol use were often difficult to maintain, and outcome trajectories varied substantially across studies. These findings suggest that brief interventions alone may be insufficient to address the multiple behavioral, psychological, and social factors associated with complex psychiatric and medical comorbidity, and that more intensive, integrated, or repeated-contact interventions may be required to achieve durable improvements.

Results among socially vulnerable populations (*n* = 3 classifications) were similarly mixed. High attrition, unstable housing, limited healthcare access, and competing psychosocial demands frequently reduced intervention engagement and follow-up participation (Rhodes et al., 2015; Thompson et al., 2017). Nevertheless, studies incorporating digital supports or structured booster contacts demonstrated some potential for improving engagement and supporting longer-term outcome maintenance in these populations (Tucker et al., 2024).

Finally, workplace, community, primary care, and other outpatient populations (*n* = 5 classifications) generally demonstrated modest but variable short-term improvements (Bogner et al., 2021; Constant et al., 2021; Harder et al., 2020; Jo et al., 2023; Kuwabara et al., 2022). Within this subgroup, short-term improvements were generally modest and variable. Across the broader evidence base, null or attenuated effects were more frequently observed in lower-intensity, pilot, or community-based interventions and were often accompanied by reduced intervention adherence, incomplete follow-up participation, or social instability (Martín-Pérez et al., 2019; Soares & Vargas, 2020; Thompson Jr et al., 2017).

Overall, the findings were broadly consistent with Hypothesis 3, suggesting that greater clinical and psychosocial complexity may be associated with more variable or less consistent intervention responses. In contrast, populations characterized by lower clinical complexity and greater intervention engagement generally demonstrated more consistent alcohol-related improvements following BMI.

### 3.6 Findings related to hypothesis 4: Comparative effectiveness according to delivery modality

Most studies (*n* = 25) primarily used face-to-face BMI delivery, whereas five incorporated hybrid or technology-assisted components and two primarily used telephone-based delivery. Face-to-face BMI delivered in clinical settings generally demonstrated greater reductions in alcohol-related outcomes compared with assessment-only, educational, or brief advice control conditions. Group-based BMI approaches demonstrated effects broadly comparable to individual delivery in community and university settings (Martín-Pérez et al., 2019; Soares & Vargas, 2020), although the number of available studies remained limited.

Among the seven studies incorporating technology-assisted or remote-delivery formats, most demonstrated short-term improvements, although fewer maintained statistically significant effects beyond 6–12 months. Technology-assisted and hybrid BMI interventions achieved modest short-term reductions in alcohol consumption generally comparable in magnitude to traditional face-to-face approaches while improving intervention accessibility and scalability (Constant et al., 2021; Gex et al., 2023; Hasin et al., 2022; Hettema et al., 2018; Hides et al., 2021; Staton et al., 2025; Stein et al., 2021). Hybrid approaches typically combined clinician-delivered BMI sessions with smartphone monitoring, text-message boosters, or web-based self-monitoring tools. Included digital interventions retained at least some clinician-guided MI components, although the degree of technological support varied substantially across studies. Technology-assisted and hybrid interventions also varied in intensity, with multi-contact or booster-supported digital formats generally demonstrating more sustained effects than minimally guided single-contact approaches. Evidence regarding sustained effectiveness beyond 6–12 months remained limited and inconsistent among technology-assisted interventions.

Overall, the findings were broadly consistent with Hypothesis 4, suggesting that technology-assisted and hybrid BMI approaches may achieve short-term effectiveness comparable to traditional face-to-face interventions, although evidence regarding long-term sustainability remains less consistent.

### 3.7 Certainty of evidence (GRADE assessment)

Using the GRADE framework, the overall certainty of evidence regarding BMI effectiveness for reducing alcohol consumption was rated as moderate (Table 5). Because all included studies were randomized controlled trials, the certainty assessment began at a high level and was subsequently downgraded for risk of bias, inconsistency, and indirectness where applicable. Downgrades were primarily related to performance bias associated with self-reported outcomes, inconsistency in long-term effectiveness, attrition in several studies, and substantial heterogeneity in participant populations, intervention formats, and outcome measures.

**Table 5:** GRADE Evidence Profile for BMI Interventions for Alcohol Use Outcomes.

Comparison	Population / Setting	Studies (Participants)	Main Outcomes	Key Findings	Sustainability	Certainty (GRADE)	Main Downgrades
Face-to-face BMI vs. usual care or brief advice	Emergency department and hospital-based populations	9 studies (≈ 2130)	Heavy drinking days, binge drinking episodes, alcohol-related consequences	Consistent short-term reductions in heavy drinking and alcohol-related harms were reported across most studies	Effects were generally strongest within the first 6 months and frequently attenuated during longer follow-up periods	Moderate	Inconsistency in long-term outcomes; performance bias associated with lack of participant blinding
BMI vs. control conditions	University and emerging adult drinkers	10 studies (≈ 1448)	Binge drinking frequency, alcohol consumption, alcohol-related consequences	Consistent improvements in binge drinking frequency and alcohol-related harms were observed across most studies	Strong short-term effectiveness; limited evidence regarding long-term maintenance	Moderate	Variability in outcome measures and limited long-term follow-up
BMI interventions	Individuals with psychiatric comorbidities or chronic medical conditions (including HIV/HCV)	10 studies (≈ 1506)	AUDIT scores, heavy drinking days, alcohol consumption	Modest but variable improvements were observed, particularly in AUDIT scores and heavy drinking frequency	Long-term maintenance was less consistent across studies	Low	Attrition, population heterogeneity, clinical complexity, and variability in intervention intensity
BMI interventions incorporating repeated contact	Mixed populations and settings	19 studies (≈ 5460)^c	Alcohol consumption, binge drinking, heavy drinking days, alcohol-related consequences	Interventions incorporating repeated contact generally demonstrated better maintenance of alcohol-related improvements than single-contact approaches	Improved maintenance beyond 6 months was more frequently reported, although findings remained heterogeneous	Moderate	Substantial heterogeneity across intervention formats; inability to isolate the independent effects of session number and booster contacts
Technology-assisted or hybrid BMI	Emerging adults, community, primary care, and outpatient populations	10 studies (≈ 1778)	Alcohol consumption, binge drinking frequency, alcohol-related harms	Short-term improvements were generally comparable to those observed in traditional face-to-face BMI interventions while increasing accessibility and scalability	Evidence regarding effectiveness beyond 6–12 months remained limited and inconsistent	Low	Marked heterogeneity in digital intervention formats, performance bias, and variable long-term engagement

Note. GRADE certainty ratings were based on the domains of risk of bias, inconsistency, indirectness, imprecision, and publication bias. Randomized controlled trials were initially rated as high-certainty evidence and downgraded when concerns were identified. Participant numbers are approximate because some studies contributed to more than one analytical category; therefore, totals across rows should not be summed. Repeated-contact interventions included multiple BMI sessions, structured booster contacts, or both.

Main reasons for downgrading: Inconsistency in long-term effectiveness, performance bias due to the lack of participant blinding, substantial attrition in complex or socially vulnerable populations, and methodological heterogeneity in participant characteristics, intervention formats, and outcome measures.

Abbreviations: AUDIT = Alcohol Use Disorders Identification Test; BMI = Brief Motivational Interviewing; HIV = Human Immunodeficiency Virus; HCV = Hepatitis C Virus.

The certainty of evidence was strongest for short-term reductions in binge drinking frequency and alcohol-related consequences, whereas evidence regarding long-term sustainability remained less consistent across studies. Evidence certainty for long-term outcomes was downgraded because of inconsistency, attrition, and variability in intervention intensity across studies. No major concerns regarding publication bias were identified, although the relatively small number of long-term studies limited certainty estimates for sustained outcomes.

## 4 Discussion

### 4.1 Overall effectiveness of BMI interventions

This systematic review synthesized evidence from 32 randomized controlled trials examining BMI interventions for alcohol misuse across heterogeneous adult populations and healthcare settings. Most included studies reported reductions in alcohol consumption, binge drinking behaviors, heavy drinking days, and alcohol-related harms following BMI interventions. However, intervention effects were not observed consistently across studies, and approximately one-quarter of the included trials did not demonstrate statistically significant improvements in primary alcohol-related outcomes. The findings therefore suggest that BMI may contribute to short-term reductions in hazardous alcohol use and alcohol-related harms among adults, although the magnitude, consistency, and durability of effects varied across populations and study contexts.

These findings further clarify factors associated with BMI effectiveness, including population complexity, intervention intensity, delivery modality, and follow-up duration. The overall pattern of findings generally supported the conceptual assumptions that guided this review, particularly regarding the short-term effectiveness of BMI, the potential value of repeated contact for maintaining improvements, and the more variable outcomes observed among clinically or socially vulnerable populations.

The present findings are broadly consistent with previous systematic reviews and meta-analyses demonstrating small to moderate, but clinically meaningful, effects of MI–based interventions for alcohol use (Foxcroft et al., 2016; Kaner et al., 2017, Kaner et al., 2018; Mun et al., 2023; Schwenker et al., 2023). Earlier syntheses similarly reported reductions in hazardous drinking and alcohol-related consequences, particularly during short-term follow-up periods (Hettema et al., 2005; Lundahl et al., 2010). The predominance of short-term benefits observed in the present review is also consistent with previous evidence indicating that MI-based interventions often produce their strongest effects within the first few months after intervention delivery, with diminishing effects over time (Kaner et al., 2018; Schwenker et al., 2023). A recent meta-analysis focusing specifically on university students likewise supported the effectiveness of MI, including BMI for reducing excessive alcohol consumption (Serrano-Fernández et al., 2025). Beyond confirming prior evidence, this review extends previous literature by focusing on contemporary trials published between 2015 and 2025 and by examining intervention intensity, delivery modality, and population complexity within a single synthesis across diverse adult populations. The included studies spanned multiple continents and healthcare settings, providing a broader perspective on the applicability of BMI across different cultural and clinical contexts.

### 4.2 Population differences and clinical complexity

The findings suggest that intervention effectiveness was not uniform across populations. Although most studies reported at least partial improvements, approximately one-quarter of the included trials failed to demonstrate statistically significant improvements in their primary alcohol-related outcomes. This finding highlights that BMI may not produce uniformly consistent benefits across all adult populations or clinical contexts. More consistent positive findings were observed among university students, emerging adults, and individuals with lower levels of psychiatric or medical comorbidity, whereas findings among participants with greater clinical or psychosocial complexity were generally more variable. These observations suggest that population complexity may influence intervention responsiveness; however, considerable heterogeneity remained across studies, and causal conclusions should be interpreted cautiously.

Younger adults may be particularly responsive to BMI because drinking behaviors are often less chronically established, ambivalence toward alcohol use may remain more modifiable, and social or developmental transitions may increase openness to behavioral change (Magill et al., 2018; Serrano-Fernández et al., 2025; Tanner-Smith & Lipsey, 2015). In contrast, intervention effects were generally more modest and less sustained among individuals with co-occurring psychiatric disorders, chronic medical conditions, or substantial social vulnerability. Similar patterns have been reported in previous MI literature, in which intervention effects tend to be attenuated among populations facing multiple psychosocial stressors or co-occurring mental health conditions (Magill et al., 2018; Saitz, 2014).

These patterns align with prior evidence suggesting stronger effects among individuals with lower baseline severity and higher readiness to change (Lundahl et al., 2010; Magill et al., 2018; Serrano-Fernández et al., 2025). In more clinically complex contexts, sustained behavior change may require more intensive, integrated, or prolonged support strategies beyond brief intervention alone in some populations (Saitz, 2014). Individuals facing co-occurring psychiatric symptoms, unstable living conditions, or chronic medical illness may encounter competing treatment demands and environmental stressors that limit the effectiveness of brief motivational approaches. These factors may reduce intervention engagement and hinder the translation of motivation into sustained behavior change.

Moreover, training clinicians on tailored BMI approaches for specific populations in contexts presenting ‘teachable moments’ may further optimize intervention effectiveness (Kehler et al., 2025). One included study also suggested comparable reductions in heavy alcohol use across racial and ethnic subgroups of emerging adults (Contreras-Pérez et al., 2024). However, this finding should be interpreted cautiously, as evidence regarding demographic moderators of BMI effectiveness remains limited and inconsistent across the broader literature (Padilla et al., 2026). Consequently, although BMI appears applicable across diverse populations, further research is needed to clarify whether demographic characteristics influence treatment response, intervention engagement, or long-term outcomes.

### 4.3 Intervention intensity and long-term sustainability

Examination of intervention characteristics revealed that studies reporting more sustained improvements were more commonly associated with multi-session interventions, booster contacts, or technology-assisted follow-up support, whereas single-session interventions more frequently demonstrated short-term benefits only. Although direct comparisons were not possible, this pattern suggests that intervention intensity may play an important role in sustaining behavioral change over time. However, because the present review was not designed to directly compare intervention intensity categories, these observations should be interpreted as descriptive patterns rather than evidence of causal superiority. Studies incorporating repeated contact more frequently reported maintenance of alcohol-related improvements beyond 6–12 months compared with studies that did not include reinforcement contacts. This observation is consistent with previous reviews suggesting that booster sessions and ongoing monitoring may enhance maintenance of treatment gains following brief alcohol interventions, although the magnitude of these effects has varied across studies (Kaner et al., 2018; Magill et al., 2018). However, the evidence remained heterogeneous, and the independent contributions of multiple BMI sessions and booster contacts could not be clearly distinguished.

The overall pattern suggests that BMI alone may be insufficient to sustain long-term behavioral change among socially vulnerable or clinically complex populations. However, these findings should be interpreted cautiously because the independent effects of session number and booster contacts could not be fully separated. Many interventions that incorporated booster contacts also involved multiple BMI sessions, making it difficult to determine which component contributed most strongly to sustained outcomes.

Nevertheless, the overall pattern suggests that repeated contact, regardless of whether delivered through additional sessions or booster follow-up contacts, may help reinforce motivation, strengthen self-efficacy, and support maintenance of behavior change over time. This interpretation is consistent with self-regulation and relapse-prevention frameworks, which emphasize ongoing reinforcement, feedback, and monitoring as important mechanisms for sustaining behavioral change after initial treatment gains (Marlatt & Donovan, 2005; Miller & Rollnick, 2023). Accordingly, the present findings support the conceptual importance of repeated contact, although the relative contributions of session number and booster contacts remain unclear.

Future studies directly comparing session frequency and booster strategies are needed to clarify their independent contributions to long-term outcomes. These findings suggest that BMI may function most effectively as an early or opportunistic intervention, whereas maintenance strategies involving repeated contacts, booster support, or digital monitoring may be necessary to sustain behavior change over time (Braitman & Henson, 2016; Tucker et al., 2024). Combining brief interventions with broader system-level support may further enhance durability (Carey et al., 2007; D'Amico et al., 2018; Zhu et al., 2024).

### 4.4 Digital and hybrid BMI delivery models

Notably, all studies categorized as multi-session plus digital interventions incorporated some form of ongoing monitoring or reinforcement, including smartphone applications, SMS reminders, telephone monitoring, or mobile follow-up support. This observation suggests that the apparent benefits of digital BMI interventions may partly reflect their capacity to provide ongoing reinforcement and monitoring rather than the digital platform itself.

A notable development in recent years is the increasing adoption of digitally delivered and hybrid BMI interventions. Technology-assisted and hybrid BMI interventions were increasingly represented within the recent literature, and most studies included in the present review reported short-term improvements in alcohol-related outcomes, although evidence regarding sustained effectiveness remained limited and inconsistent. These findings are broadly consistent with previous research suggesting that telehealth, smartphone applications, text messaging, and blended intervention models can reduce alcohol consumption and alcohol-related harms while improving intervention accessibility and scalability (Bertholet et al., 2023; Boß et al., 2018; Riper et al., 2018). Such approaches may be particularly valuable for younger adults and socially vulnerable populations who encounter barriers to traditional face-to-face services (Dedert et al., 2015; Muench et al., 2017, Muench et al., 2024).

However, previous studies have suggested that fully unguided digital interventions generally produce smaller and less durable effects than clinician-supported or hybrid formats (Baumann et al., 2021; Chau et al., 2024; Cucciare et al., 2021; Cunningham et al., 2017). Because fully unguided interventions were excluded from the present review, their comparative effectiveness could not be evaluated directly. Among the clinician-guided and hybrid BMI interventions included in this review, technology-assisted approaches generally achieved short-term benefits comparable to those of traditional face-to-face interventions, although evidence regarding long-term sustainability remained less consistent. These findings suggest that minimal but meaningful clinician involvement may remain important for preserving key motivational processes during digital intervention delivery (Graham et al., 2020; Grekin et al., 2024).

### 4.5 Mechanisms of action and teachable moments

The beneficial effects of BMI may be partially explained by core MI processes, including enhancement of intrinsic motivation, resolution of ambivalence, and strengthening of self-efficacy (Magill et al., 2014, Magill et al., 2017, Magill et al., 2019; Miller & Rollnick, 2023). These mechanisms may be particularly relevant during brief clinical encounters where rapid engagement and readiness for change are critical to intervention success. In emergency departments and acute healthcare settings, BMI may capitalize on “teachable moments” following alcohol-related injuries, intoxication-related visits, or other acute health events, during which individuals may be more receptive to behavioral change (D'Onofrio et al., 2008; D'Onofrio & Degutis, 2010). Recent evidence further suggests that MI-based interventions, including BMI, may not only reduce substance use behaviors but also enhance treatment motivation and psychological well-being among individuals with substance use disorders, although these outcomes were not systematically evaluated in the present review (Kayaoğlu & Tanrıkulu, 2025; Padilla et al., 2026; Tan et al., 2023).

### 4.6 Strengths and limitations

This review has several strengths, including its focus on recent randomized controlled trials, inclusion of heterogeneous populations, intervention structures, and delivery modalities, and rigorous application of PRISMA, RoB 2, and GRADE methodologies. Furthermore, the included studies were conducted across diverse geographical regions, including North America, Europe, Asia, Africa, and South America, and involved a wide range of populations, including university students, emergency department patients, individuals living with HIV, persons with psychiatric comorbidities, and socially vulnerable groups. This diversity enhances the external relevance of the findings while also providing insight into BMI effectiveness across multiple clinical contexts. Nevertheless, several limitations should be acknowledged. Considerable clinical and methodological heterogeneity limited direct comparability across studies. The absence of meta-analytic pooling also limited the ability to estimate overall effect sizes quantitatively because of substantial heterogeneity in intervention formats, outcome measures, and follow-up durations. Most outcomes relied on self-reported alcohol measures, which may be subject to recall and social desirability bias. Attrition rates varied considerably across studies, ranging from approximately 9% to 38% at final follow-up, with higher loss to follow-up generally observed among socially vulnerable populations and participants with psychiatric comorbidities. In addition, approximately one-third of the included studies did not report formal motivational interviewing fidelity assessments, limiting confidence regarding the consistency of intervention implementation across studies.

Another limitation is that potentially important addictive comorbidities, such as tobacco use, cannabis use, stimulant use, or polysubstance use, were not systematically examined across the included studies. Because co-occurring substance use disorders may influence treatment engagement, relapse risk, and responsiveness to BMI interventions, unmeasured addictive comorbidity may have contributed to variability in intervention outcomes. Additional heterogeneity arose from differences in intervention providers, which ranged from physicians and psychologists to nurses, counselors, social workers, and lay health workers. Variability in training, experience, and MI competence may have influenced intervention delivery and treatment outcomes. Furthermore, although studies from Europe, Asia, Africa, and South America were represented, the evidence base remained disproportionately derived from high-income Western countries, particularly the United States, which may limit the generalizability of findings to other healthcare systems and cultural contexts.

Finally, the overall certainty of evidence was rated as moderate using the GRADE framework, primarily because of risk of bias associated with self-reported outcomes, inconsistency in long-term findings, attrition, and heterogeneity across studies. Consequently, the conclusions of this review should be interpreted with appropriate caution.

### 4.7 Clinical implications and future directions

From a clinical perspective, BMI may be particularly useful when delivered during periods of increased readiness to change, such as following alcohol-related medical encounters or during routine healthcare contacts. Given its relatively brief format and adaptability across settings, BMI may represent a practical first-line intervention for hazardous alcohol use. However, for individuals with substantial psychiatric, medical, or social complexity, BMI may be most effective when integrated with ongoing monitoring, booster contacts, or broader treatment services.

Future research should prioritize standardized alcohol-related outcome measures, longer follow-up periods, and direct comparisons of intervention intensity, delivery modality, and maintenance strategies. Additional research is needed to identify which patient populations are most likely to benefit from specific BMI approaches and to clarify the relative contributions of clinician involvement, booster contacts, and technology-assisted support in sustaining long-term outcomes.

Overall, the available evidence suggests that BMI is a flexible and potentially clinically meaningful intervention for reducing hazardous alcohol use and alcohol-related harms, particularly during short-term follow-up periods. However, the moderate certainty of evidence and variability across studies indicate that treatment effects should be interpreted with appropriate caution. Moreover, variation in outcomes across populations and intervention formats suggests that sustained effectiveness may be influenced by intervention intensity, ongoing engagement, and adaptation to the clinical and psychosocial needs of specific populations.

## 5 Conclusion

BMI appears to be a flexible and clinically meaningful intervention for reducing hazardous alcohol use and alcohol-related harms across diverse adult populations and healthcare settings. The evidence synthesized in this review suggests that BMI is generally associated with short-term reductions in alcohol consumption, binge drinking behaviors, heavy drinking days, and related adverse consequences. However, intervention effects were not consistently sustained beyond 6–12 months and appeared to be more variable among individuals with psychiatric comorbidities or substantial social vulnerability. The findings further suggest that repeated contact, booster sessions, digital monitoring, or ongoing clinician involvement may help support maintenance of behavior change over time. Given the moderate certainty of the available evidence and the substantial heterogeneity across studies, these conclusions should be interpreted with appropriate caution. Future research should directly compare session frequency, booster strategies, digital support approaches, and clinician involvement to determine their independent and combined contributions to sustaining long-term alcohol-related outcomes across different population subgroups.

## CRediT authorship contribution statement

**Mei-Hsin Ho:** Writing – review & editing, Writing – original draft, Methodology, Investigation, Formal analysis, Data curation, Conceptualization. **Jui-Hsiu Tsai:** Writing – review & editing, Validation, Resources, Investigation, Data curation. **Chizimuzo T C Okoli:** Writing – review & editing, Supervision, Methodology. **Rei-Mei Hong:** Writing – review & editing, Visualization, Validation, Software, Project administration.

## Funding

This research received no external funding.

## Declaration of competing interest

The authors declare that they have no known competing financial interests or personal relationships that could have appeared to influence the work reported in this paper.

## Data Availability

Data will be made available on request.

## Appendix A Supplementary data

Supplementary material 1 Database-Specific Search Strategies.

Supplementary material 2 Summary of BMI Effects on Alcohol Outcomes Across Follow-up Periods.
