# Supplementary material for: Effectiveness of brief motivational interviewing for alcohol misuse: a systematic review of randomized controlled trials
Source: Addict Behav Rep. 2026 Jul 10;24:100727. doi: 10.1016/j.abrep.2026.100727 (PMC13382408; doi:10.1016/j.abrep.2026.100727)
Supplement: Supplementary file 1 — Supplementary material 1 Database-Specific Search Strategies. [file mmc1.docx]

## Supplementary Material Table S1. Database-Specific Search Strategies

| PubMed | (“motivational interviewing”[MeSH Terms] OR “motivational interviewing” OR “brief motivational interviewing” OR “brief intervention” OR BMI)  AND  (“alcohol drinking”[MeSH Terms] OR alcohol OR drinking OR ethanol OR “alcohol use disorder” OR “heavy drinking”)  AND  (“2015/01/01”[Date - Publication] : “2025/12/31”[Date - Publication]) |
| --- | --- |
| PsycINFO | (motivational interviewing OR brief motivational interviewing OR brief intervention OR BMI)  AND  (alcohol OR drinking OR ethanol OR alcohol use disorder OR heavy drinking))  Limiters: Peer-reviewed journal articles, English language, Published 2015–2025, Adults (18+) |
| CINAHL | (MH “Motivational Interviewing” OR “motivational interviewing” OR “brief intervention”)  AND  (MH “Alcohol Drinking+” OR alcohol OR drinking OR ethanol OR “alcohol use disorder”) |
| Web of Science | TS=(motivational interviewing OR brief motivational interviewing OR brief intervention OR BMI)  AND  TS=(alcohol OR drinking OR ethanol OR alcohol use disorder OR heavy drinking)  Refined by: Article, English, Published 2015–2025 |

**Note**: The search was designed to maximize sensitivity by excluding comparator and outcome terms. Full details of study selection and data extraction are described in the Methods section. A total of 32 studies met the inclusion criteria.
