# Supplementary material for: Effectiveness of brief motivational interviewing for alcohol misuse: a systematic review of randomized controlled trials
Source: Addict Behav Rep. 2026 Jul 10;24:100727. doi: 10.1016/j.abrep.2026.100727 (PMC13382408; doi:10.1016/j.abrep.2026.100727)
Supplement: Supplementary file 2 — Supplementary material 2 Summary of BMI Effects on Alcohol Outcomes Across Follow-up Periods. [file mmc2.docx]

## Supplementary Material Table S2. Summary of BMI Effects on Alcohol Outcomes Across Follow-up Periods

| **Outcome** | **Short-term (≤3 months)** | **Medium-term (>3–6 months)** | **Long-term (>6–12 months)** | **Interpretation** |
| --- | --- | --- | --- | --- |
| Weekly alcohol consumption | Frequently improved | Moderate maintenance | Often attenuated or non-significant | Improvements were most commonly observed among university students and emergency department populations; however, effects on overall alcohol consumption volume tended to weaken over time |
| Binge drinking frequency | Consistently improved | Frequently maintained | Moderate maintenance | This was one of the most consistently improved alcohol-related outcomes across heterogeneous populations and study settings |
| Heavy drinking days | Frequently improved | Frequently maintained | Variable maintenance | More sustained reductions were generally observed in interventions incorporating repeated contact, although the independent contributions of multiple sessions and booster contacts could not be determined |
| AUDIT scores | Consistently improved (typically 2–5 points) | Moderate maintenance | Often attenuated | Short-term improvements were reported across several populations, including studies involving psychiatric and medical comorbidities, although long-term maintenance was less consistent |
| Alcohol-related consequences | Consistently improved | Frequently maintained | Relatively better maintenance | Reductions in alcohol-related consequences demonstrated the greatest durability and appeared less susceptible to attenuation than alcohol consumption measures |

**Note.** This table summarizes outcome patterns descriptively and is intended for illustrative purposes only. No meta-analysis was performed; therefore, the findings should not be interpreted as pooled effect estimates. *Repeated contact* refers to interventions incorporating multiple BMI sessions, booster contacts, or technology-assisted reinforcement and monitoring.
